# Non-synonymous single-nucleotide variations of the human oxytocin receptor gene and autism spectrum disorders: a case–control study in a Japanese population and functional analysis

**DOI:** 10.1186/2040-2392-4-22

**Published:** 2013-07-01

**Authors:** Wen-Jie Ma, Minako Hashii, Toshio Munesue, Kenshi Hayashi, Kunimasa Yagi, Masakazu Yamagishi, Haruhiro Higashida, Shigeru Yokoyama

**Affiliations:** 1 Department of Biophysical Genetics, Kanazawa University Graduate School of Medicine, 13-1 Takara-machi, Kanazawa 920-8640, Japan; 2 Research Center for Child Mental Development, Kanazawa University, Kanazawa 920-8640, Japan; 3 Department of Clinical Laboratory, Kanazawa University Hospital, Kanazawa 920-8641, Japan; 4 Department of Internal Medicine, Kanazawa University Graduate School of Medicine, Kanazawa 920-8640, Japan; 5 Core Research for Evolutional Science and Technology (CREST), Japan Science and Technology Agency, Tokyo 102-0075, Japan; 6 MEXT Strategic Research Program for Brain Sciences (SRPBS), Okazaki 444-0840, Japan

**Keywords:** Autism spectrum disorders, Inositol-1,4,5-trisphosphate, Intracellular free calcium, Oxytocin, Oxytocin receptor, Single-nucleotide variation

## Abstract

**Background:**

The human oxytocin receptor (hOXTR) is implicated in the etiology of autism spectrum disorders (ASDs) and is a potential target for therapeutic intervention. Several studies have reported single-nucleotide polymorphisms (SNPs) of the *OXTR* gene associated with ASDs. These SNPs, however, reside outside the protein-coding region. Not much is known about genetic variations that cause amino acid substitutions that alter receptor functions.

**Methods:**

Variations in the *OXTR* gene were analyzed in 132 ASD patients at Kanazawa University Hospital in Japan and 248 unrelated healthy Japanese volunteers by re-sequencing and real-time polymerase chain reaction-based genotyping. Functional changes in variant OXTRs were assessed by radioligand binding assay and measurements of intracellular free calcium concentrations ([Ca^2+^]_i_) and inositol 1,4,5-trisphosphate (IP_3_) levels.

**Results:**

Six subjects (4.5%) in the ASD group and two in the control group (0.8%) were identified as heterozygotes carrying the R376G variation (rs35062132; c.1126C>G); one individual from the ASD group (0.8%) and three members of the control group (1.2%) were found to be carrying R376C (c.1126C>T). The C/G genotype significantly correlated with an increased risk of ASDs (odds ratio (OR) = 5.83; 95% confidence interval (CI) = 1.16 to 29.33; *P* = 0.024, Fisher’s exact test). Consistently, the G allele showed a correlation with an increased likelihood of ASDs (OR = 5.73; 95% CI = 1.15 to 28.61; *P* = 0.024, Fisher’s exact test). The frequencies of the C/T genotype and the T allele in the ASD and control groups did not differ significantly. We also examined changes in agonist-induced cellular responses mediated by the variant receptors hOXTR-376G and hOXTR-376C. OXT-induced receptor internalization and recycling were faster in hOXTR-376G-expressing HEK-293 cells than in cells expressing hOXTR-376R or hOXTR-376C. In addition, the elevation in [Ca^2+^]_i_ and IP_3_ formation decreased in the cells expressing hOXTR-376G and hOXTR-376C tagged with enhanced green fluorescent protein (EGFP), in comparison with the cells expressing the common-type hOXTR-376R tagged with EGFP.

**Conclusions:**

These results suggest that the rare genetic variation rs35062132 might contribute to the pathogenesis of ASDs, and could provide a molecular basis of individual differences in OXTR-mediated modulation of social behavior.

## Background

Oxytocin (OXT) is a nonapeptide hormone primarily produced by magnocellular neurons in the paraventricular and supraoptic nuclei of the hypothalamus [1-6]. Oxytocin is secreted into the systemic circulation via the hypothalamic-neurohypophyseal system and transported to exert its peripheral effects, such as uterine contractions and milk ejection [1-6]. Oxytocin is released into the brain from dendrites [7] or centrally projected axonal terminals [8], and it acts on various areas of the central nervous system, modulating affiliative and social behaviors, emotional states, and cognitive functions in many animal species [1-6].

The actions of OXT are mediated by the OXT receptor (OXTR), which belongs to the GTP-binding protein-coupled receptor family [9]. It is well established that the OXTR is coupled to heterotrimeric G_q/11_ protein and that it stimulates phospholipase C_β_ (PLC_β_), leading to the production of inositol 1,4,5-trisphosphate (IP_3_) and 1,2-diacylglycerol (DAG) [9]. Inositol 1,4,5-trisphosphate induces an increase in intracellular calcium concentration ([Ca^2+^]_i_) by triggering IP_3_ receptor-mediated Ca^2+^ release from intracellular stores, whereas DAG activates protein kinase C (PKC), which phosphorylates downstream signaling molecules [9]. Upon prolonged agonist stimulation, OXTR undergoes desensitization and internalization through phosphorylation by GPCR kinase 2 (GRK2) and subsequent interaction with β-arrestin 2 [9-13]. The internalized OXTRs are then recycled to the cell surface via vesicles containing small GTPases, Rab4 and Rab5 [13].

Previous studies have shown that impaired OXTR-mediated signaling causes behavioral abnormalities [2,6,14]. Mice deficient in the *OXTR* gene show diminished social discrimination, increased aggressive behavior, reduced cognitive flexibility, and increased susceptibility to seizures [15,16]. Using homozygous mutant mice lacking CD38, a transmembrane protein with ADP-ribosyl cyclase activity, we have shown that a decrease in the formation of cyclic ADP-ribose (cADPR) results in dysfunction of Ca^2+^-induced Ca^2+^ release for OXT secretion in hypothalamic neurons. The reduction in cADPR levels also causes marked defects in maternal nurturing and social behaviors similar to those observed in *OXT*- and *OXTR*-knockout mice [17,18]. We also demonstrated that ADP-ribosyl cyclase activity increases after OXTR stimulation, regulating OXT release in the hypothalamus and pituitary in adult male mice [19].

In humans, OXTR has been implicated in the pathogenesis of neuropsychiatric disorders and considered a potential target for therapeutic intervention [20]. Gregory *et al*. [21] have reported a heterozygous deletion of the *OXTR* gene, located on chromosome 3p25, in a patient with autism whose mother had a putative obsessive-compulsive disorder. In addition, many studies have demonstrated that single-nucleotide polymorphisms (SNPs) of the *OXTR* gene are associated with autism spectrum disorders (ASDs), social anxiety disorders, and schizophrenia [22-29]. However, all SNPs reported so far reside outside the protein-coding region, and their functional importance is unclear. If allelic variations leading to amino acid substitutions in the *OXTR* gene were associated with these neuropsychiatric disorders, then such variations would help to predict the etiological relevance based on protein structure-function relationship and to introduce individualized OXT-based therapy.

Therefore, we analyzed nucleotide variations in the protein-coding regions of the human *OXTR* gene in ASD patients and unrelated healthy controls. Furthermore, we focused on a triallelic variation (rs35062132; c.1126C> G/T; the nomenclature of variation recommended by the Human Genome Variation Society [30]) and investigated whether the amino acid substitution R376G/C causes any changes in OXTR-mediated cellular responses.

## Methods

### Participants

We recruited 132 ASD subjects (102 males, 30 females; 15.9 ± 0.7 years) from the outpatient psychiatry department of the Kanazawa University Hospital. All subjects fulfilled the DSM-IV criteria for pervasive developmental disorder. The diagnoses were made by two experienced child psychiatrists through interviews and clinical record reviews as described previously [31], and the subjects had no apparent physical anomalies. The two experienced child psychiatrists independently confirmed the diagnosis of ASD for all patients by semi-structured behavior observation and interviews with the subjects and their parents. During each of these interviews with parents, which were helpful in the evaluation of autism-specific behaviors and symptoms, the examiners used one of the following methods: the Asperger Syndrome Diagnostic Interview [32], the Autism Diagnostic Interview-Revised (ADI-R)[33], the Pervasive Developmental Disorders Autism Society Japan Rating Scale [34], the Diagnostic Interview for Social and Communication Disorders [35], or the Tokyo Autistic Behavior Scale [36]. Based on these evaluation methods, 97 patients were classified as autistic disorder (autism), 10 as Asperger’s disorder, and 25 as pervasive developmental disorder not otherwise specified (PDD-NOS). The 248 controls (143 males, 105 females; 31.3 ± 0.6 years) were unrelated healthy Japanese volunteers. All patients and controls were Japanese with no non-Japanese parents or grandparents. This study was approved by the ethics committees of Kanazawa University School of Medicine. All examinations were performed after informed consent according to the Declaration of Helsinki.

### Re-sequencing

Genomic DNA was extracted from venous blood samples using the Wizard Genomic DNA Purification kit (Promega, Madison, WI, USA), or from nails using the ISOHAIR DNA extraction kit (Nippon Gene, Tokyo, Japan). DNA fragments containing exons 3 and 4 of the hOXTR gene were amplified by PCR in a 20-μl reaction mixture containing 2.5 ng genomic DNA, 200 μM dNTPs, 200 nM of each primer, 4 μl Ampdirect G/C (Shimadzu, Kyoto, Japan), 4 μl Ampdirect-4 (Shimadzu), and 1 unit *Ex Taq* DNA polymerase (Takara Shuzo, Otsu, Japan). The amplification procedure consisted of denaturation at 96°C for 1 min, followed by 35 cycles of 96°C for 30 s, 65°C for 1 min, and 72°C for 1 min. The primers used were FWD1 or FWD4 and REV1 for exon 3, and FWD5 and REV3 for exon 4 (sequences are given in Table 1). After amplification, PCR products were purified with the High Pure PCR Cleanup Micro kit (Roche, Mannheim, Germany), subjected to cycle sequencing reaction (BigDye version 1.1; Applied Biosystems, Foster City, CA, USA) according to the manufacturer’s protocol, and analyzed on an ABI PRISM 310 Genetic Analyzer (Applied Biosystems). The primers used were FWD1–FWD5 and REV1–REV6 (Table 1).

**Table 1:** Oligonucleotides used in this study

**Nucleotide sequencing**	**Real-time PCR-based genotyping**		**Site-directed mutagenesis**	**EGFP tagging**
	**Amplification**	**TaqMan MGB probes**		
FWD1: TGGACTCAGCAGATCCGTCCG	FWD8: GCTCCGCCAGCTACCTGAAG	376G-FAM: TGAGCCATGGCAGCTCC	R376G-FWD: CCTTTGTCCTGAGCCATGGCAGCTCCAGCCAGAGG	EGFP-FWD: CCGCAGGTGCACATCTTCTC
FWD2: CTAAGCATCGCCGACCTGGT	REV7: TGGTGGGTCACGCCGTGGAT	376R-VIC: TGAGCCATCGCAGCTCC	R376G-REV: CCTCTGGCTGGAGCTGCCATGGCTCAGGACAAAGG	EGFP-REV: GTGGATCCCGCCGTGGATGG
FWD3: CCGCAGGTGCACATCTTCTC		376C-FAM: TGAGCCATTGCAGCTCC	R376C-FWD: CCTTTGTCCTGAGCCATTGCAGCTCCAGCCAGAGG	
FWD4: TGGAGTCTCCAGGAGTGGA			R376C-REV: CCTCTGGCTGGAGCTGCAATGGCTCAGGACAAAGG	
FWD5: GTCTGGAAGTGGCTCCAGTG				
REV1: CCTGGACATTCTGAGGCAGC				
REV2: GATGAGCTTGACGCTGCTGAC				
REV3: GTCAGCAGCGTCAAGCTCATC				
REV4: CAGTCGAAGACGCCGTC				
REV5: ACATGAGCAGCAGCAGG				
REV6: CAGGAGCAGGATGAGAC				

All sequences are given in the 5' to 3' direction. Nucleotides for rs35062132 are underlined. *C,* cysteine; *FWD,* forward; *G,* glycine; *MGB,* minor groove binder; *R,* arginine; *REV*, reverse.

### Real-time PCR

Two primers, FWD8 and REV7 (Table 1), were designed to amplify a 138-bp product encompassing rs35602132 at the mRNA position 1748 (g.8734707; c.1126). Three custom-designed TaqMan minor groove binder (MGB) probes were obtained from Applied Biosystems: 376G-FAM and 376C-FAM targeted to the variant alleles, and 376R-VIC to the common allele (Table 1). PCR was performed in 20-μl mixtures containing 10 μl TaqMan universal master mix (Applied Biosystems), 200 nM of each primer, 100 nM 376R-FAM, 100 nM 376G-VIC or 376C-VIC, and 10 ng of sample DNA. Thermocycling was performed on the ViiA 7 Real-Time PCR System (Applied Biosystems). The amplification was conducted at 60°C for 30 s, 95°C for 5 min, and 40 cycles of 95°C for 15 s and 60°C for 1 min. Data were analyzed with SDS2.3 software (Applied Biosystems).

### Site-directed mutagenesis

cDNAs encoding hOXTR variants at amino acid residue 376 were generated by site-directed mutagenesis as described previously [37]. pCHOXTR [38] harboring hOXTR-376R, a common-type receptor, was used as a template for PCR; the primers used were R376G-FWD and R376G-REV (Table 1) for the R376G substitution, and R376C-FWD and R376C-REV (Table 1) for R376C. The amplified products were treated with *EX Taq* DNA polymerase (Takara Shuzo) at 72°C for 10 min and cloned into a pCR2.1-TOPO vector (Invitrogen, Carlsbad, CA, USA) to yield pCHOXTR-376G and pCHOXTR-376C. The cDNA inserts were verified by nucleotide sequencing.

### Construction of expression plasmids

The 1.4-kb *Eco*RI fragments from pCHOXTR [38], pCHOXTR-376G, and pCHOXTR-376C were cloned into the *Eco*RI site of the mammalian expression plasmid pcDNA3.1 (+) (Invitrogen) to yield pcDNAHOXTR-376R, pcDNAHOXTR-376G, and pcDNAHOXTR-376C, respectively.

In some experiments, hOXTRs of the common type and variants were fused to the enhanced green fluorescent protein (EGFP). Expression plasmids for these fusion proteins were constructed essentially as described previously [38]. In brief, the 1.4-kb *Eco*RI fragments obtained from pCHOXTR [38], pcDNAHOXTR-376G, and pcDNAHOXTR-376C were subjected to PCR using primers EGFP-FWD and EGFP-REV (Table 1). The amplified fragments were cleaved with *Pst*I and *Bam*HI. The resulting 0.46-kb *Pst*I (1334)/*Bam*HI (primer) fragments and the 0.78-kb *Bam*HI (556)/*Pst*I (1334) fragment from pCHOXTR were ligated to *Bam*HI/*Bgl*II-cleaved pEGFP-N3 (Clontech) to yield pHOXTR-376R-EGFP, pHOXTR-376G-EGFP, and pHOXT-R376C-EGFP. Restriction endonucleasesites are identified by numbers (in parentheses; in accordance with the data deposited in GenBank under accession number NM_000916) indicating the 5'-terminal nucleotide generated by cleavage.

### Cell culture and transfection

Human embryonic kidney HEK-293 cells and COS-7 cells were maintained in DMEM supplemented with 10% FBS at 37°C in a humidified atmosphere of 95% air and 5% CO_2_. NG108-15 neuroblastoma × glioma hybrid cells were cultured as described previously [39]. Cells were grown in culture dishes to 80 to 90% confluence and transfected with expression plasmids using FuGENE HD Transfection Reagent (Roche) following the manufacturer’s instruction.

### Radioligand binding assay

COS-7 cells transfected with pcDNAHOXTR-376R, pcDNAHOXTR-376G, pcDNAHOXTR-376C, or pcDNA3.1 (+) were collected 48 h after transfection, and resuspended in homogenization buffer (25 mM Tris, pH 7.4, 1 mM EDTA, 250 mM sucrose). The cells were homogenized and centrifuged at 1,500 *g* at 4°C for 10 min, to pellet nuclei and intact cells. The resulting supernatants were centrifuged at 40,000 *g* at 4°C for 30 min, and crude membrane pellets were suspended in binding buffer containing 50 mM HEPES (pH 7.4), 5 mM MgCl_2_, 1 mM CaCl_2_, and 0.2% BSA. Protein concentration was determined by the bicinchoninic acid assay (Pierce, Rockford, IL, USA) using BSA as a standard. [Tyrosyl-^3^H]-oxytocin ([^3^H]-OXT; PerkinElmer Life Sciences, Waltham, MA, USA) was diluted in the binding buffer to concentrations of 0.05 to 4 nM. Specific binding assays were performed in 5-μg cell membrane preparations incubated at room temperature for 1 h with increasing concentrations of [^3^H]-OXT, in the absence or presence of 200-fold excess of unlabeled OXT. Binding reaction mixtures were filtered through a GF/C glass fiber filter (Whatman, Maidstone, Kent, UK) and washed three times with Wash Buffer (50 mM HEPES, pH 7.4, 500 mM NaCl, 0.1% BSA). Radioactivity associated with membranes retained by glass filters was quantified in a liquid scintillation counter (LSC-5100; Aloka, Tokyo, Japan). Specific binding was calculated by subtracting non-specific binding in the presence of 200-fold excess of OXT at each radioligand concentration from total binding. Affinity (*K*_d_) and maximal binding capacity (*B*_max_) of saturation binding were obtained from saturation isotherm specific binding data by nonlinear regression curve analysis using the standard equation for a rectangular hyperbola fitted to one site with the GraphPad Prism 5 (GraphPad Software Inc., San Diego, CA, USA). Competition binding experiments were performed in duplicate by incubating 5 μg of cell membranes at room temperature for 1 h, in the same medium with 1 nM [^3^H]-OXT and increasing concentrations of unlabeled OXT (10^−12^ to 10^−5^ M). Log IC_50_ values were derived from nonlinear least-squares analysis using the GraphPad Prism 5 software (GraphPad Software Inc.).

### Measurement of receptor recycling

HEK-293 cells were plated on poly-D-ornithine-coated glass coverslips and cultured overnight. After transfection with pcDNAHOXTR-376R, pcDNAHOXTR-376G, or pcDNAHOXTR-376C, the cells were incubated at 37°C in serum-free DMEM for different times, in the absence or presence of OXT (100 nM). Reactions were stopped by removing the medium and fixing the cells with 4% paraformaldehyde at room temperature for 10 min. The cells were then blocked with PBS containing 1% BSA and 1.5% normal horse serum for 30 min, and incubated with goat anti-OXTR antibody (N-19; 1:200; sc-8103, Santa Cruz Biotechnology, Santa Cruz, CA, USA) at 4°C overnight. After washing in PBS, the cells were incubated with donkey anti-goat IgG (H + L) antibody conjugated with Alexa Fluor 488 (1:500; Invitrogen) combined with 4',6-diamidino-2-phenylindole (DAPI; 1 μg/ml; Dojindo, Kumamoto, Japan). In some experiments, pSNAP_f_-ADRβ2 (New England Biolabs, Ipswich, MA, USA), an expression plasmid for β2 adrenergic receptor fused to SNAP_f_, was cotransfected into HEK-293 cells. The cells were labeled with SNAP-Surface Alexa Fluor 488 (2 μM; New England Biolabs), fixed with 4% paraformaldehyde, and reacted with goat anti-OXTR antibody (N-19; 1:200; Santa Cruz) at 4°C overnight, followed by incubation with donkey anti-goat IgG (H + L) antibody conjugated with Alexa Fluor 594 (1:500; Invitrogen); none of the solutions contained any cell-permeabilizing reagents. Fluorescent images were obtained using confocal laser scanning microscopes (LSM5 Pascal; Carl Zeiss, Jena, Germany; FluoView FV10i, Olympus). Data were analyzed using the FV10-ASW software (Olympus).

### [Ca^2+^]_i_ measurement

We measured [Ca^2+^]_i_ using the fluorescent Ca^2+^ indicator fura-2-acetoxymethyl ester (fura-2/AM). HEK-293 cells or NG108-15 cells transfected with pHOXTR-376R-EGFP, pHOXTR-376G-EGFP, or pHOXTR-376C-EGFP were loaded with fura-2/AM to a final concentration of 3 μM in complete medium and incubated at 37°C. After 30-min loading, the cells were washed three times with HEPES-buffered saline (HBS) solution (145 mM NaCl, 5 mM KCl, 1 mM MgCl_2_, 20 mM HEPES-NaOH, 2 mM CaCl_2_, 20 mM glucose, pH 7.4). Cells expressing EGFP-tagged OXTRs were visualized at a wavelength of 488 nm before OXT application. The fluorescence of the cells loaded with fura-2/AM was then measured at 37°C, at the determined sites, through a pinhole (10 to 20 μm in diameter). We used alternating excitation wavelengths of 340 and 380 nm in a Ca^2+^ microspectrofluorometric system (OSP-3 Model; Olympus, Tokyo, Japan), as described previously [39]. The Ca^2+^ emission was detected every 10 s for 5 min after OXT application. The ratio of fluorescence at 340 nm and 380 nm (*F*_340_*/F*_380_) was used to determine [Ca^2+^]_i_. All data were normalized to the baseline fluorescence (*F*_0_) recorded 10 s before OXT addition, and given as *F/F*_0*.*_

### IP_3_ accumulation assay

HEK-293 cells were plated at a density of approximately 5 × 10^4^ per well in a 24-well plate, and transiently transfected with pcDNAHOXTR-376R, pcDNAHOXTR-376G or pcDNAHOXTR-376C, or pcDNA3.1(+). Before treatment with OXT, the transfected cells were washed and incubated in HBS containing 2 mM CaCl_2_, 10 mM glucose, and 10 mM LiCl at 37°C for 10 min. After OXT treatment, the reaction was terminated at designated time points by the addition of 50 μl of 10% (w/v) perchloric acid. The acid-soluble extract was neutralized with 150 μl of 1.53 M KOH-75 nM HEPES, and perchloric acid was precipitated on ice and removed by a brief centrifugation. The IP_3_ aqueous extract was then examined with an Inositol-1,4,5-triphosphate [^3^H]-Radio Receptor Assay Kit (PerkinElmer Life Sciences).

### Statistical analyses

Data obtained from genetic studies were analyzed using a contingency table and the Fisher’s exact test (GraphPad Prism 5; GraphPad Software Inc.). All data from the *in vitro* studies are shown as mean ± standard error of the mean. Statistical significance was determined by the Student’s *t *test or two-way analysis of variance (ANOVA) using an interactive fitting program (GraphPad Prism 5; GraphPad Software Inc.); *P* values smaller than 0.05 were considered to be statistically significant.

## Results

### Genetic analysis

**Figure 1:**
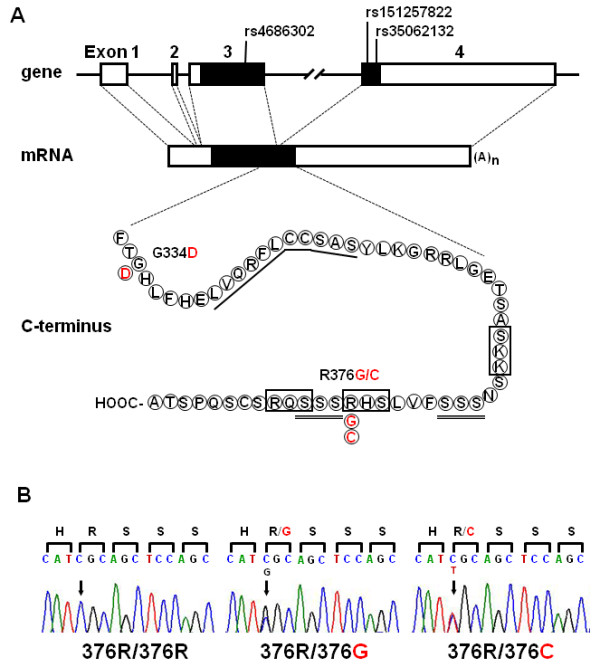
**Triallelic variation rs35062132 in the human *OXTR *gene. (A)** Structure of the human *OXTR* gene and amino acid sequence of the *C*-terminal portion of the human OXTR. White and black blocks represent untranslated regions and protein-coding regions, respectively, and red letters represent minor amino acid variations. Indicated in the protein-coding region are rs4686302, rs151257822, and rs35062132, in which allelic heterozygosity was detected in this study. The region required for coupling to Gq isunderlined; potential PKC phosphorylation sites are indicated by boxes, and Ser triplets are double underlined. **(B)** Representative electrophoretograms of the rs35062132 (c.1126C>G/T; R376G/C). Left, homozygote for the common allele (*376R/376R*); middle, heterozygous carrier of c.1126C>G (376R/376G); right, heterozygous carrier of c.1126C>T (376R/376C). Arrows indicate the polymorphic position. Nucleotides and amino acids for minor alleles are shown in red.

As the entire amino acid sequence of the hOXTR is encoded by exons 3 and 4, as illustrated in Figure 1A, we initially re-sequenced both exons for genetic variations. Of 27 single-nucleotide variations listed in the NCI dbSNP database, we detected minor alleles of rs4686302, rs151257822, and rs35062132 in 59 ASD patients and 30 unrelated healthy Japanese volunteers (Figure 1A, Table 2). As c.1126 G>C (rs35062132; R376G) was detected in two ASD patients but not in controls (Table 2, Figure 1B), we selected rs35062132 for further analysis.

**Table 2:** **Frequency of non-synonymous single-nucleotide variations in the *OXTR *gene detected by re-sequencing**

**dbSNP-ID**^**a**^	**mRNA**^**b**^	**Allele**	**Amino acid**	**Amino acid**	**Number of cases**	
	**position**		**position**	**residue**	**ASD ( *n *= 59)**	**Control ( *n *= 30)**
rs237908	19	T/G	7	S/A	T/T (0) T/G (0) G/G (59)	T/T (0) T/G (0) G/G (30)
rs237907	40	T/G	14	S/A	T/T (0) T/G (0) G/G (59)	T/T (0) T/G (0) G/G (30)
rs237906	46	T/G	16	S/A	T/T (0) T/G (0) G/G (59)	T/T (0) T/G (0) G/G (30)
rs189386	64	T/G	22	W/G	T/T (0) T/G (0) G/G (59)	T/T (0) T/G (0) G/G (30)
rs113718500	65	C/G	22	A/G	C/C (0) C/G (0) G/G (59)	C/C (0) C/G (0) G/G (30)
rs237903	86	T/C	29	V/A	T/T (0) T/C (0) C/C (59)	T/T (0) T/C (0) C/C (30)
rs171114	188	G/C	63	G/A	G/G (0) G/C (0) C/C (59)	G/G (0) G/C (0) C/C (30)
rs143644523	220	T/C	74	F/L	T/T (0) T/C (0) C/C (59)	T/T (0) T/C (0) C/C (30)
rs138770371	316	G/T	106	D/Y	G/G (0) G/T (0) T/T (59)	G/G (0) G/T (0) T/T (30)
rs115324487	515	C/T	172	A/V	C/C (0) C/T (0) T/T (59)	C/C (0) C/T (0) T/T (30)
rs150746704	616	G/C	206	V/L	G/G (0) G/C (0) C/C (59)	G/G (0) G/C (0) C/C (30)
**rs4686302**	652	A/G	218	T/A	A/A (3) A/G(12) G/G (34)	A/A (1) A/G(14) G/G (15)
rs143908202	661	A/G	221	S/G	A/A (0) A/G (0) G/G (59)	A/A (0) A/G (0) G/G (30)
rs145921539	684	T/G	228	C/W	T/T (0) T/G (0) G/G (59)	T/T (0) T/G (0) G/G (30)
rs184175311	700	G/A	234	E/K	G/G (0) G/A (0) A/A (59)	G/G (0) G/A (0) A/A (30)
rs61740241	712	A/G	238	T/A	A/A (0) A/G (0) G/G (59)	A/A (0) A/G (0) G/G (30)
rs151141371	755	C/G	252	A/G	C/C (0) C/G (0) G/G (59)	C/C (0) C/G (0) G/G (30)
rs139854982	760	T/C	254	C/R	T/T (0) T/C (0) C/C (59)	T/T (0) T/C (0) C/C (30)
rs144366756	764	G/T	255	G/V	G/G (0) G/T (0) T/T (59)	G/G (0) G/T (0) T/T (30)
rs237901	818	T/C	273	M/T	T/T (0) T/C (0) C/C (59)	T/T (0) T/C (0) C/C (30)
rs144814761	841	A/G	281	M/V	A/A (0) A/G (0) G/G (59)	A/A (0) A/G (0) G/G (30)
rs140488139	963	A/C	321	K/N	A/A (0) A/C (0) C/C (59)	A/A (0) A/C (0) C/C (30)
**rs151257822**	1001	A/G	334	D/G	A/A (0) A/G (0) G/G (59)	A/A (0) A/G (1) G/G (29)
rs143927655	1015	A/G	339	K/E	A/A (0) A/G (0) G/G (59)	A/A (0) A/G (0) G/G (30)
rs148899442	1100	G/A	367	S/N	G/G (0) G/A (0) A/A (59)	G/G (0) G/A (0) A/A (30)
rs144289031	1121	A/G	374	N/S	A/A (0) A/G (0) G/G (59)	A/A (0) A/G (0) G/G (30)
**rs35062132**	1126	G/C	376	G/R	G/G (0) G/C (2) C/C (57)	G/G (0) G/C (0) C/C (29)
**rs35062132**	1126	T/C	376	C/R	T/T (0) T/C (0) C/C (57)	T/T (0) T/C (1) C/C (29)

^a^IDs are from dbSNP of the National Center for Biotechnology Information [40]. Bold IDs indicate the presence of allelic heterozygosity. ^b^A of the ATG of the initial methionine is denoted nucleotide +1.

We genotyped an additional 73 ASD patients and 218 healthy volunteers for rs35062132 variation using the TaqMan-based real-time PCR method. In total, we obtained data from 132 ASD patients and 248 controls. Six individualsin the ASD group (4.5%) and two in the control group (0.8%) were identified as heterozygotes carrying the R376G variation (rs35062132; c.1126C>G). One person in the ASD group (0.8%) and three in the control group (1.2%) carried R376C (c.1126C>T; Table 3). No homozygous carriers for the minor alleles were observed. The *χ*^2^ goodness-of-fit test showed that the genotype frequency distribution of rs35062132 did not deviate from Hardy-Weinberg equilibrium in the patient group (*χ*^2^ = 0.098, *P* = 0.99) or the control group (*χ*^2^ = 0.026, *P* = 1.00). The genotype and allele frequencies of rs35062132 obtained from all participants are summarized in Table 3. The C/G genotype was significantly correlated with an increased risk of ASDs (odds ratio (OR) = 5.83; 95% confidence interval (CI) = 1.16 to 29.3; *P* = 0.024, Fisher’s exact test; Table 3). Consistently, the G allele also showed a correlation with an increased likelihood of ASDs (OR = 5.73, 95% CI = 1.15 to 28.6; *P* = 0.024, Fisher’s exact test; Table 3). The frequencies of the C/T genotype and the T allele did not significantly differ between the ASD and control groups (Table 3).

**Table 3:** Comparison of the genotype and allele frequencies of rs35062132 at Kanazawa University Hospital for ASDs

	**Cases**	**Control**	**Odds ratio**	***P***
	**(*n *= 132)**	**(*n *= 248)**	**(95% CI)**	
**Genotype**				
C/C	125 (94.7%)	243 (98.0%)	Referent	
C/G	6 (4.5%)	2 (0.8%)	5.83 (1.16,29.3)	0.024
C/T	1 (0.8%)	3 (1.2%)	0.65 (0.07,6.30)	1.00
G/G	0	0		
T/T	0	0		
G/T	0	0		
**Allele**				
C	257 (97.3%)	491 (99.0%)	Referent	
G	6 (2.3%)	2 (0.4%)	5.73 (1.15,28.6)	0.024
T	1 (0.4%)	3 (0.6%)	0.64 (0.07,6.16)	1.00

*CI,* confidence interval. *P* values obtained by Fisher’s exact test are given.

### Radioligand binding properties of the common and variant hOXTRs

To find out whether the amino acid changes at the residue 376 affect the receptor functions, we compared ligand-binding properties of the common and variant hOXTRs. In saturation binding and Scatchard analysis of ^3^[H]-OXT binding to membranes prepared from COS-7 cells transfected with hOXTR-expression plasmids, the variants hOXTR-376G and hOXTR-R376C, as well as the common-type hOXTR-376R, exhibited single high-affinity binding sites. Values of *K*_d_ were 0.90 ± 0.28, 1.1 ± 0.12, and 1.2 ± 0.19 nM (*n* = 15 in each group), and values of *B*_max_ were 3153 ± 531, 3202 ± 234, and 3120 ± 339 fmol/mg of protein (*n* = 15 in each group) for hOXTR-376R, hOXTR-376G, and hOXTR-376C, respectively. Competition binding experiments confirmed that the variant and common receptors did not differ significantly: values of IC_50_ were 1.05 ± 0.47, 0.49 ± 0.23, and 0.71 ± 0.30 nM (*n* = 15 in each group) for hOXTR-376R, hOXTR-376G, and hOXTR-376C, respectively. These data indicate that both variations have no or little effect on OXT binding.

### Agonist-induced receptor internalization and recycling in HEK-293 cells

We then examined the agonist-induced internalization and recycling of hOXTRs in HEK-293 cells, which have been most extensively used for investigation of GPCR internalization and trafficking. HEK-293 cells transfected with an expression plasmid for hOXTR-376R, hOXTR-376G, or hOXTR-376C were stimulated with 100 nM OXT for 5, 10, 15, 30, 45, 60, 90, and 120 min. At each time point, the cells were fixed and stained with antibody pecific for the amino (*N*)-terminus, located extracellularly, of the OXTR under impermeable conditions. The specificity of the staining was confirmed by the comparison with the staining of mock-transfected and non-transfected HEK-293 cells (Additional file 1: Figure S1).

**Figure 2:**
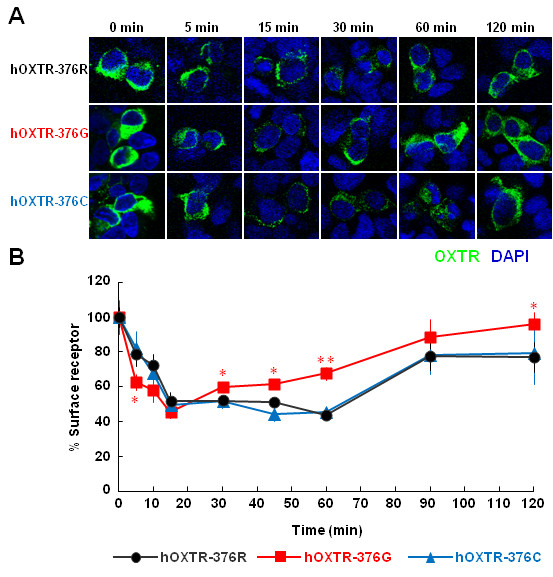
**Agonist-induced internalization and recycling of common and variant hOXTRs. (A)** Fluorescent images of HEK-293 cells transfected with expression plasmids for the common-type hOXTR-376R and variants hOXTR-376G and hOXTR-R376C. Cells were double-labeled using antibody against the *N*-terminus of hOXTR (Alexa Fluor 488, green) and a nuclear marker (DAPI, blue). Images were obtained by confocal laser scanning microscopy at the indicated time points after the addition of OXT (100 nM). **(B)** Fluorescence intensities for hOXTRs were quantified and expressed as the percentage of receptors remaining at the cell surface. Data are mean ± standard error of the mean (*n* = 15 to 30 cells from five cultures). Statistical significance was evaluated by the Student’s *t* test (* *P* <0.05, **P <0.01; compared with hOXTR-376R).

Immunoreactivity for hOXTRs was visualized by confocal laser scanning microscopy (Figure 2A). Immunofluorescence was measured and expressed as a percentage of the intensity for receptors remaining at the cell surface. Cell-surface intensity for hOXTR-376R reached the lowest value of 43.9 ± 2.3% (*n* = 30) after 60 min, and then gradually increased to 77.1 ± 8.7% (*n* = 29) of the prestimulatory level at 120 min. OXT-induced internalization in the cells expressing hOXTR-376C were very similar to that for hOXTR-376R: the surface intensity decreased to 45.1 ± 3.3% (*n* = 30) at 60 min, and then elevated to 79.4 ± 18.1% (*n* = 8) at 120 min. In contrast, the surface intensity of hOXTR-376G showed a significantly faster decrease than that for hOXTR-376R at 5 min after OXT application (62.5 ± 4.7% (*n* = 25) for hOXTR-376G, 78.7 ± 6.7% (*n* = 15) for hOXTR-376R; *P* <0.05; Student’s *t* test). In addition, hOXTR-376G showed significantly higher levels of surface intensities from 30 min to 120 min after OXT application (Figure 2B). The values at 30, 45, 60, and 120 min were 59.6 ± 2.7 (*n* = 30, *P* <0.05; Student’s *t* test), 61.4 ± 3.0 (*n* = 30, *P* <0.05; Student’s *t* test), 67.7 ± 3.7 (*n* = 30, *P* <0.01; Student’s *t* test), and 96.1 ± 6.6% (*n* = 23, *P* <0.05; Student’s *t* test) for hOXTR-376G, compared with 52.0 ± 2.5 (*n* = 30), 51.1 ± 3.1 (*n* = 30), 43.9 ± 2.3 (*n* = 30), and 77.1 ± 8.7% (*n* = 29) for hOXTR-376R, respectively (Figure 2B). The majority of the hOXTR-immunoreactivity was colocalized with fluorescence for SNAP_f_-tagged β2-adrenergic receptor as a plasma membrane marker (>84%; Additional file 2: Figure S2). These data demonstrate that, upon agonist stimulation, hOXTR-376G is internalized into the cytoplasm and recycled to the plasma membrane more rapidly than hOXTR-376R and hOXTR-376C.

### Agonist-induced Ca^2+^ mobilization in HEK-293 cells and NG108-15 neuronal cells

**Figure 3:**
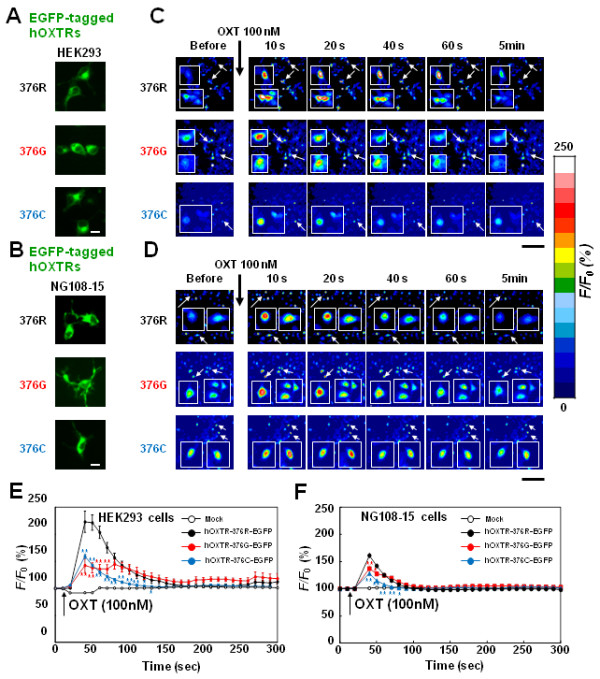
**Agonist-induced Ca**^**2+ **^**mobilization. (A,B)** HEK-293 cells and NG108-15 neuronal cells were transfected with expression plasmids for EGFP-tagged hOXTR-376R (common type) and visualized by fluorescence microscopy. Scale bar, 10 μm. **(C,D)** Images of fluorescence changes in HEK-293 cells **(C)** and NG108-15 cells **(D)** expressing hOXTR-376R-EGFP (top), hOXTR-376G-EGFP (middle), and hOXTR-376C (bottom) are shown before and after treatment with 100 nM OXT. The ratio of fluorescence intensities detected at 340 and 380 nm was used to determine [Ca^2+^]_i_. Emission ratio values ((*F*/*F*_0_) were pseudo-colored to represent calcium concentrations according to the scale bar on the right. Black arrows indicate application with 100 nM OXT; and white arrows show large [Ca^2+^]_i_ changes in the cells after stimulation. Insets show larger images of the cells indicated by arrows. Scale bar, 100 μm. **(E,F)** Average time courses of changes in [Ca^2+^]_i_ elicited with 100 nM OXT were measured in HEK-293 cells **(E)** and NG108-15 neuronal cells **(F)** expressing hOXTR-376R-EGFP, hOXTR-376G-EGFP, or hOXTR-376C-EGFP and in mock-transfected cells. Values are mean ± standard error of the mean (*n* = 24 to 30 cells from three or four independent cultures; six to ten cells were analyzed in each culture). Statistical significance was evaluated by the Student’s *t* test (* *P* <0.05, **P <0.01; compared with hOXTR-376R-EGFP).

To examine whether the amino acid substitutions altered the coupling to intracellular signaling system, we measured OXT-induced Ca^2+^ mobilization. Both HEK-293 cells and NG108-15 neuroblastoma × glioma hybrid cells were transfected with an expression plasmid for EGFP-tagged hOXTR-376R, hOXTR-R376G, or hOXTR-376C. Before OXT stimulation, EGFP fluorescence was homogenously distributed in both HEK-293 cells and NG108-15 neuronal cells expressing hOXTR-376R-EGFP, hOXTR-376G-EGFP, and hOXTR-376C-EGFP, as previously reported [13] (Figure 3A,B). After loading these cells with Ca^2+^-sensitive fluorescent dye fura-2/AM, the effect of the amino acid variations on OXT-induced increase in [Ca^2+^]_i_ was analyzed using a dual-wavelength (340 and 380 nm) fluorescence spectrophotometer.

The addition of 100 nM OXT to HEK-293 cells expressing hOXTR-376R-EGFP induced a rapid initial increase of [Ca^2+^]_i_; the levels reached 223 ± 19.6% (*n* = 24) of the prestimulatory level and then returned to the basal level within 3 min (Figure 3C,E). In contrast, both hOXTR-376G-EGFP- and hOXTR-376C-EGFP-expressing HEK-293 cells exhibited significantly smaller initial peaks: the maximum [Ca^2+^]_i_ was 142 ± 13.2% (*n* = 24) and 159 ± 3.8% (*n* = 31) for hOXTR-376G-EGFP and hOXTR-376C-EGFP, respectively (Figure 3C,E). Mock-transfected control cells did not show Ca^2+^ mobilization at all (Figure 3C,E).

The same tendency was observed in NG108-15 neuronal cells. [Ca^2+^]_i_ initially increased to relatively low levels compared with those in HEK-293 cells. The maximum [Ca^2+^]_i_ levels at the initial peak in hOXTR-376G-EGFP- and hOXTR-376C-EGFP-expressing NG108-15 cells were 137 ± 5.1% (*n* = 28) and 128 ± 2.0% (*n* = 18), respectively, significantly lower than those in hOXTR-376R-EGFP-expressing cells, which reached 161 ± 5.1% (*n* = 24; Figure 3D,F).

### Agonist dose-dependent Ca^2+^ mobilization

**Figure 4:**
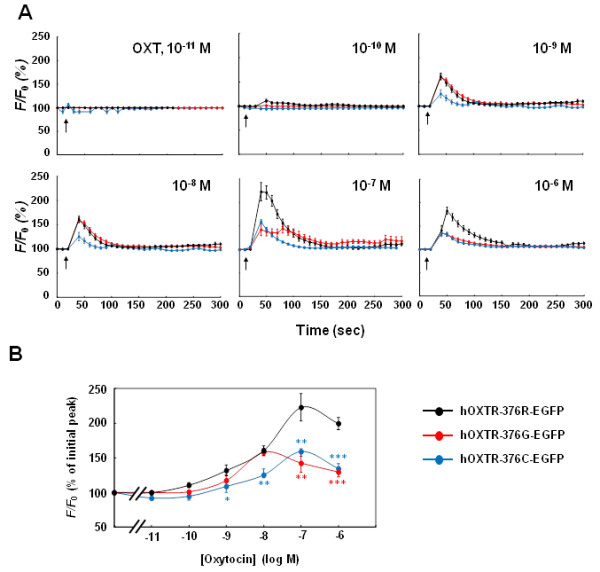
**Agonist dose-dependent Ca**^**2+ **^**mobilization. (A)** The changes in [Ca^2+^]_i_ induced by various concentrations of OXT were measured in HEK-293 cells expressing hOXTR-376R-EGFP, hOXTR-376G-EGFP, or hOXTR-376C-EGFP. **(B)** Relationship between OXT concentrations and [Ca^2+^]_i_ at the initial peak. Cells were infused with the indicated concentration of OXT. Each plot shows a percentage of [Ca^2+^]_i_ at the initial peak after treatment with OXT. Data are mean ± standard error of the mean (*n* = 8 to 30 cells from three or four independent cultures; two to eight cells were analyzed in each culture). Statistical significance was evaluated by the Student’s *t* test (* *P* <0.05, **P <0.01; compared with hOXTR-376R-EGFP).

We further examined the OXT dose-dependence during the increase in [Ca^2+^]_i_ in HEK-293 cells expressing the common and variant hOXTRs. OXT concentrations ranging from 10^−11^ to 10^−6^ M were tested. When the OXT concentration was lower than 10^−10^ M, no differences in the initial [Ca^2+^]_i_ rise were observed in the cells expressing hOXTR-376R-EGFP, hOXTR-376G-EGFP, and hOXTR-376C-EGFP (Figure 4A,B). In the cells expressing hOXTR-376R-EGFP, the maximum initial increase in [Ca^2+^]_i_ was at 10^−7^ M OXT (Figure 4A,B).

In HEK293 cells expressing hOXTR-376G-EGFP, the maximal [Ca^2+^]_i_ increase was obtained at 10^−8^ M OXT (158 ± 5.2% (*n* = 24); Figure 4A,B). OXT at 10^−7^ and 10^−6^ M resulted in significantly smaller [Ca^2+^]_i_ increases compared with those for hOXTR-376R-EGFP, with initial peak values being 142 ± 13.2% (*n* = 24) and 129 ± 7.2% (*n* = 16), respectively.

In HEK-293 cells expressing hOXTR-376C-EGFP, the maximal [Ca^2+^]_i_ increase was obtained at 10^−7^ M (159 ± 3.8% (*n* = 31); Figure 4A,B). At concentrations higher than 10^−9^ M, the OXT-induced increase in [Ca^2+^]_i_ was significantly reduced (Figure 4A,B). These data suggested that the rs35062132 variations (R376G/C) might alter the OXTR-mediated changes in the intracellular calcium concentrations.

### Agonist-induced IP_3_ accumulation in HEK-293 cells

**Figure 5:**
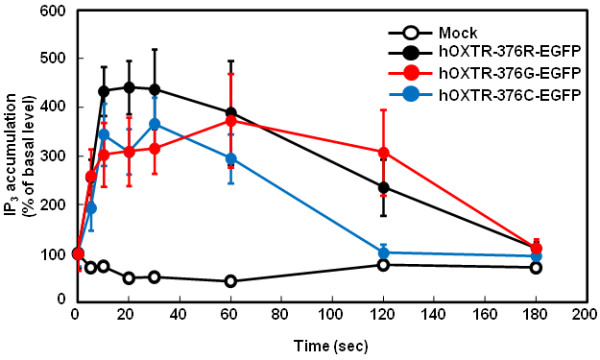
**Agonist-induced IP**_**3 **_**accumulation.** Average time courses of changes in IP_3_ accumulation elicited with 100 nM OXT; IP_3_ levels were measured in HEK293 cells expressing hOXTR-376R-EGFP, hOXTR-376G-EGFP, and hOXTR-376C-EGFP. Data are expressed as mean ± standard error of the mean (*n* = 13 to 15 cultures from five independent experiments). During the initial 5 to 30 s after OXT application, a significant drop from the maximum IP_3_ levels was observed in these cells (*P* = 0.0210, *F*(1, 107) = 5.49 for hOXTR-376G-EGFP; *P* = 0.0209, *F*(1, 103) = 5.5 for hOXTR-376C-EGFP; compared with the values for hOXTR-376R-EGFP; two-way ANOVA).

IP_3_ is known to mediate OXT-induced increase in [Ca^2+^]_i_ levels. Therefore, we investigated whether the amino acid substitutions could affect IP_3_ production. As shown in Figure 5, in HEK-293 cells expressing hOXTR-376R-EGFP, 100 nM OXT elicited an increase in IP_3_ level within initial 10 s; this increase was maintained for 30 s and the level returned to its basal value after 3 min. Mock-transfected cells did not show any changes. The cells expressing hOXTR-376G-EGFP and hOXTR-376C-EGFP also stimulated IP_3_ production. During the whole period of 3 min after OXT application, IP_3_ production in cells expressing hOXTR-376G-EGFP was not statistically different compared with hOXTR-376R-EGFP (*P* = 0.1705, F(1, 217) = 1.89; two-way ANOVA), whereas cells expressing hOXTR-376C-EGFP showed significantly smaller IP_3_ production (*P* = 0.003, F(1, 210) = 9.01; two-way ANOVA). During the initial 5 to 30 s after OXT application, a slight but significant drop from the maximum IP_3_ levels was observed in these cells, compared with the values for hOXTR-376R-EGFP (*P* = 0.0210, F(1, 107) = 5.49 for hOXTR-376G-EGFP; *P* = 0.0209, F(1, 103) = 5.5 for hOXTR-376C-EGFP; two-way ANOVA).

## Discussion

The main findings of this study are as follows. (i) Triallelic non-synonymous variation (rs35062132, c.1126C>G/T; R376G/C) is observed in both ASD patients and healthy controls in a Japanese population; the frequencies of the G allele are significantly higher in the ASD patients than in healthy controls. (ii) In HEK-293 cells expressing hOXTR-376G, the agonist-induced internalization and recycling of the OXTR are faster than that in the cells expressing hOXTR-376C or hOXTR-376R. (iii) In both HEK-293 cells and NG108-15 neuronal cells, the agonist-induced increase in [Ca^2+^]_i_ mediated by hOXTR-376G-EGFP or hOXTR-376C-EGFP is smaller than the increase associated with hOXTR-376R-EGFP. These results provide new insights into the genetic architecture and therapeutic aspects of ASDs.

The present study is unique in associating non-synonymous allelic variations of the *OXTR* gene with ASDs. These variations at rs35062132 have not been reported in other disorders. To date, several studies have found ASD-associated SNPs in the *OXTR* gene, which reside in introns (rs4564970 [41], rs237897 [25], rs53576 [23,24], rs2254298 [23-25], rs2268493 [42], rs7632287 [42], rs11720238 [41]), 3'-untranslated region (rs1042778) [42], and intergenic regions (rs7632287 and rs11720238) [41]. The functional consequences of these SNPs remain unclear. Importantly, all minor alleles of these SNPs have a frequency more than 5%, and thus can be classified as common variations. In contrast, the frequencies of the G and T alleles of rs35062132 are 0.4% and 0.6%, respectively, in controls; these minor alleles can be categorized as rare [43-45]. Therefore, most previous studies favor the common disease–common variant model, in which most of the risk is caused by common genetic variations (>5% allele frequency), each allele conferring a slight risk [43-47]. By contrast, our results support the common disease–rare variant model, in which the risk is mostly attributable to rare variations (<5% allele frequency), each variant conferring a moderate but readily detectable increase in the relative risk [43-47]. In agreement with this model, we observed that the minor alleles clearly affected OXT-induced cellular responses. Future studies will be conducted to test whether behavioral changes caused by these variations are moderate and reasonably deleterious. Also it will be interesting to examine whether the rs35062132 variations cosegregate with the risk alleles in the non-coding region [22-24,41,42].

The limitation of this study is that sample size is not sufficient to analyze the rare variant alleles at rs35062132. Future studies with larger sample size or family-based association testing are necessary to conclude that the G allele of the rs35062132 is a genetic risk factor for ASDs. The result reported here should be replicated in independent populations with various ethnic backgrounds.

The amino acid variation R376G/C is located in the intracellular carboxy (*C*)-terminal tail of the receptor protein, which is critical for desensitization, internalization, and recycling of the OXTR, as in many other GPCRs [9-13]. Arg^376^ is associated with two structural components involved in these processes: (i) Arg^376^ is a part of a PKC consensus motif (Ser^374^-His^375^-Arg^376^; Figure 1A), which is thought to interact with PKC after OXT stimulation [10]; and (ii) Arg^376^ is in conjunction with one of the two Ser triplets in the *C*-terminus (Figure 1A) that are primary sites of agonist-induced phosphorylation and β-arrestin-2 binding [11]. When either one of the two Ser triplets was mutated to alanine residues, the stability of the β-arrestin-2-OXTR complex was altered, and the ability of β-arrestin-2 to internalize with the receptor was eliminated [11]. In the vasopressin receptor V2, which is highly homologous to OXTR in structure, the equivalent Ser triplets function as a retention motif. GRK phosphorylation of these Ser residues promotes the formation of stable receptor-β-arrestin complexes, followed by cotrafficking and colocalization of the complexes to endocytotic vesicles and slow recycling to the cell surface [48]. Therefore, it is suggested that the R376G substitution in the OXTR might suppress the phosphorylation of Ser residues in the Ser triplets or reduce the stability of β-arrestin binding to the triplets, resulting in faster receptor recycling.

We demonstrated that, both in HEK-293 cells and in NG108-15 neuronal cells, agonist-induced [Ca^2+^]_i_ elevation mediated by variant receptors, hOXTR-376G-EGFP and hOXTR-376C-EGFP, is smaller than that by the common receptor hOXTR-376R-EGFP. Despite the marked reduction in the peak [Ca^2+^]_i_, the decrease in IP_3_ levels was slight. Provided that Arg^376^ is not located in the known site for G_q_ binding (Figure 1A) [49], the G_q/11_/PLC_β_/IP_3_-independent, unidentified signaling pathway for [Ca^2+^]_i_ elevation might be affected by the R376G/C substitution.

Ca^2+^ signaling pathways in neurons have been implicated in the pathogenesis of ASDs [50]. However, the involvement of OXTR-mediated, PLC/IP_3_-dependent Ca^2+^ signaling in ASDs is still largely unknown. Recently, Ninan [51] has demonstrated that U73122, a PLC inhibitor, reduced OXT-induced suppression of glutamatergic synaptic transmission in pyramidal neurons of the medial prefrontal cortex, possibly by inhibiting PLC-dependent increase in postsynaptic calcium. Given that this process is critical for the OXT effects on social cognition, it is conceivable that the OXTR variations may serve as a risk factor for ASDs.

We have previously demonstrated that cADPR, which may act as an intracellular second messenger downstream of the OXTR, activates Ca^2+^ release from intracellular stores through the ryanodine receptor Ca^2+^ release channel. cADPR also initiates Ca^2+^ influx through melastatin-related transient receptor potential 2 (TRPM2) channels [38]. Our previous SNP analysis has suggested that R140W substitution of CD38 protein, a regulator of ryanodine receptor-mediated Ca^2+^-induced Ca^2+^ release for OXT secretion [17], could be a potential risk factor for a subset of Japanese ASD patients [31]. Although this study shares a part of the ASD patients with our previous analysis [31], the 140W allele of CD38 has not been found in the patients carrying the OXTR-376G or OXTR-376C until now (unpublished data). It is therefore conceivable that the underlying signaling process affected by the R376G/C substitution is independent of the CD38-mediated OXT release from hypothalamic neurons [17].

Besides their relevance to the etiology of ASDs, the results presented here might contribute to the development of new pharmacological treatments. Genetic polymorphisms of many drug targets predict responsiveness to drugs [52,53]. It is likely that the alteration in the cellular functions mediated by the variant OXTRs could cause some individual differences in both behavioral and non-behavioral responses to OXT. Although no side effects specific to intranasal OXT administration have been reported [54], new clinical information regarding functional OXTR variants will be helpful in the determination of individual administration protocols to maximize therapeutic benefit with least adverse effects.

## Conclusions

Our analysis of the genetic variation rs35062132 in the human *OXTR* gene may be important for both etiological and therapeutic aspects of ASDs. However, the results reported here should be reproduced in an independent population. Further studies with a larger sample size using subjects of different ethnicities are required to confirm that the G allele of the rs35062132 is a genetic risk factor for ASDs. Identifying rare functional variants of the OXTR will shed new light on the genetic architecture of ASDs and allow personalized pharmacological intervention using OXT.

## Abbreviations

ANOVA: Analysis of variance; ASD: Autism spectrum disorder; BSA: Bovine serum albumin; cADPR: cyclic ADP-ribose; CI: Confidence interval; CREST: Core Research for Evolutional Science and Technology; DAPI: 4',6-diamidino-2-phenylindole; DMEM: Dulbecco’s modified Eagle’s medium; EGFP: Enhanced green fluorescent protein; FBS: Fetal bovine serum; fura-2/AM: Fura-2-acetoxymethyl ester; hOXTR: Human oxytocin receptor; IP3: Inositol-1,4,5-trisphosphate; MGB: Minor groove binder; OR: Odds ratio; OXT: Oxytocin; OXTR: Oxytocin receptor; PCR: Polymerase chain reaction; PKC: Protein kinase C; SNP: Single-nucleotide polymorphism.

## Competing interests

The authors declare they have no competing interests.

## Authors’ contributions

HH and SY conceived and designed the research. WJM, TM, KH, KY, MY, and SY performed the genetic analysis of the ASD patients and healthy volunteers. WJM, MH, and SY carried out the experiments on cultured cells. WJM, HH, and SY analyzed data. WJM and SY drafted the initial manuscript; and WJM, TM, HH, and SY prepared successive versions of the document. All authors read and approved the final manuscript.

## Supplementary Material

Additional file 1: Figure S1 Antibody validation. Fluorescent images of HEK-293 cells transfected with expression plasmids for the common-type hOXTR-376R (top, left) and variants hOXTR-376G (top, middle) and hOXTR-376C (top, right). Images of HEK-293 cells transfected with an empty vector (Mock) and non-transected cells (−). Cells were stained with anti-hOXTR antibody and visualized with Alexa Fluor 488-conjugated secondary antibody (green). DAPI was used to stain cell nuclei (blue). Note that hOXTR-immunoreactivity is detected in cells transfected with expression plasmids for the common and variant hOXTRs but not in mock- and non-transfected cells. hOXTR-immunoreactive cells were detected in 11.8% (177/1500 DAPI-positive cells in total of eight different fields), 15.5% (188/1210 in total of eight different fields), 16.6% (224/1353 in total of eight different fields), 0% (0/789 in total of four different fields), and 0% (0/748 in total of four different fields) in hOXTR-376R-, hOXTR-376G-, hOXTR-376C-, mock-, and non-transfected cells, respectively. Scale bar, 20 μm. Click here for file

Additional file 2: Figure S2 Co-staining of hOXTRs with β2-adrenergic receptor as a cell-surface marker. **(A)** Fluorescent images of HEK-293 cells transfected with an expression plasmid for hOXTR-376R (left), hOXTR-376G (middle), or hOXTR-376C (right), together with that for the plasma membrane marker SNAP_f_-tagged β2 adrenergic receptor. Cells were stained with anti-hOXTR antibody and visualized with Alexa Fluor 594-conjugated secondary antibody (hOXTR, red). SNAP_f_-tagged β2 adrenergic receptor was labeled with SNAP-Surface Alexa Fluor 488 (SNAP_f_-ADRβ2, green). DAPI was used to stain cell nuclei (DAPI, blue). Note that hOXTR-immunoreactivity is mostly overlapped with fluorescence for SNAP_f_-tagged β2 adrenergic receptor (Merge, yellow). Scale bar, 10 μm*.***(B)** OXTR-immunoreactivity overlapped with SNAP_f_-tagged β2 adrenergic receptor. Data are mean ± standard error of the mean. The overlapping at 0, 30, 60 min after OXT application (100 nM) was estimated to be 86.0 ± 1.1% (*n* = 20), 83.1 ± 1.5% (*n* = 18), and 85.5 ± 1.0% at 60 min (*n* = 20), respectively, for hOXTR-376R; 86.1 ± 1.0% (*n* = 23), 84.9 ± 1.6% (*n* = 13), 84.6 ± 1.4% (*n* = 20), respectively, for hOXTR-376G; and 85.2 ± 1.2% (*n* = 32), 84.9 ± 1.8% (*n* = 16), and 84.1 ± 2.2% (*n* = 19), respectively, for hOXTR-376C. Click here for file
