# Supplementary figures and images for: Non-synonymous single-nucleotide variations of the human oxytocin receptor gene and autism spectrum disorders: a case–control study in a Japanese population and functional analysis
Source: Mol Autism. 2013 Jul 1;4:22. doi: 10.1186/2040-2392-4-22 (PMC3707786; doi:10.1186/2040-2392-4-22)

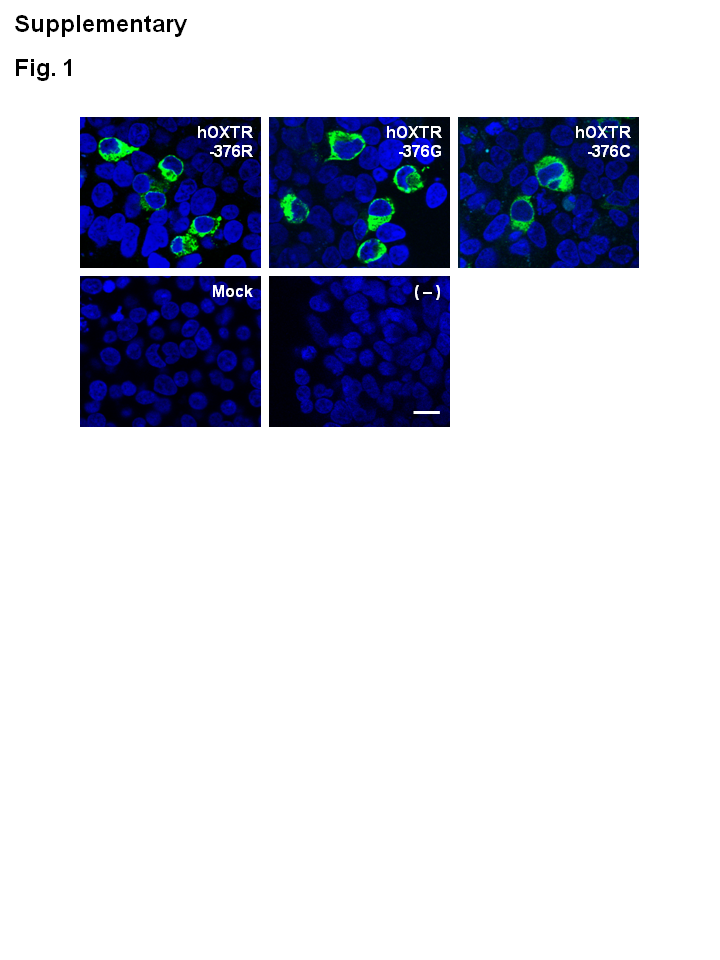

Supplement: Additional file 1: Figure S1 — Antibody validation. Fluorescent images of HEK-293 cells transfected with expression plasmids for the common-type hOXTR-376R (top, left) and variants hOXTR-376G (top, middle) and hOXTR-376C (top, right). Images of HEK-293 cells transfected with an empty vector (Mock) and non-transected cells (−). Cells were stained with anti-hOXTR antibody and visualized with Alexa Fluor 488-conjugated secondary antibody (green). DAPI was used to stain cell nuclei (blue). Note that hOXTR-immunoreactivity is detected in cells transfected with expression plasmids for the common and variant hOXTRs but not in mock- and non-transfected cells. hOXTR-immunoreactive cells were detected in 11.8% (177/1500 DAPI-positive cells in total of eight different fields), 15.5% (188/1210 in total of eight different fields), 16.6% (224/1353 in total of eight different fields), 0% (0/789 in total of four different fields), and 0% (0/748 in total of four different fields) in hOXTR-376R-, hOXTR-376G-, hOXTR-376C-, mock-, and non-transfected cells, respectively. Scale bar, 20 μm. [file 2040-2392-4-22-S1.tiff]

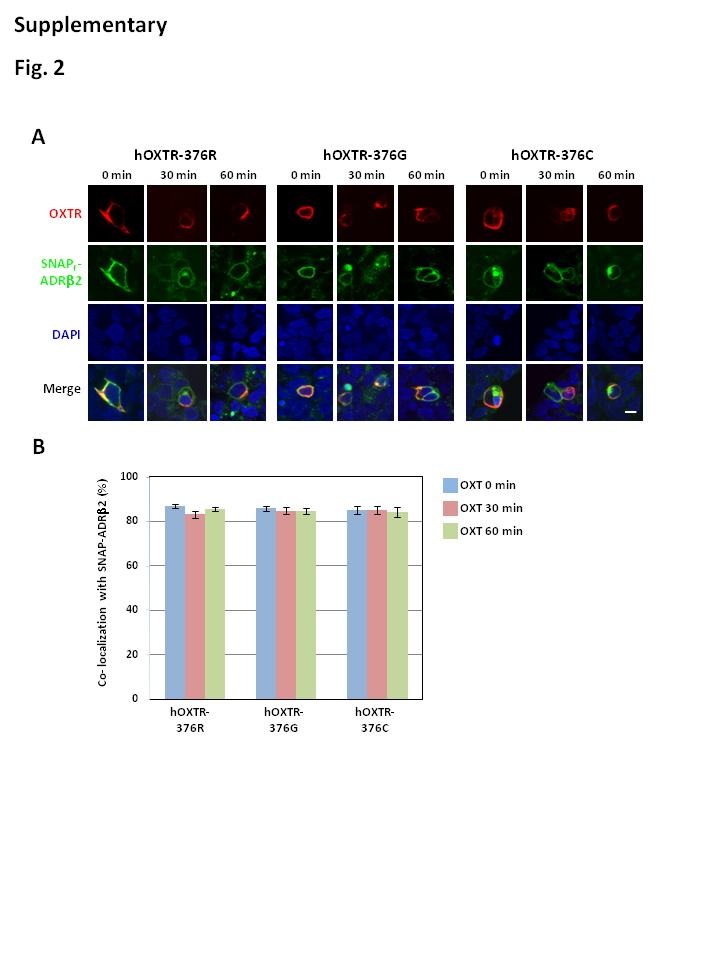

Supplement: Additional file 2: Figure S2 — Co-staining of hOXTRs with β2-adrenergic receptor as a cell-surface marker. (A) Fluorescent images of HEK-293 cells transfected with an expression plasmid for hOXTR-376R (left), hOXTR-376G (middle), or hOXTR-376C (right), together with that for the plasma membrane marker SNAPf-tagged β2 adrenergic receptor. Cells were stained with anti-hOXTR antibody and visualized with Alexa Fluor 594-conjugated secondary antibody (hOXTR, red). SNAPf-tagged β2 adrenergic receptor was labeled with SNAP-Surface Alexa Fluor 488 (SNAPf-ADRβ2, green). DAPI was used to stain cell nuclei (DAPI, blue). Note that hOXTR-immunoreactivity is mostly overlapped with fluorescence for SNAPf-tagged β2 adrenergic receptor (Merge, yellow). Scale bar, 10 μm.(B) OXTR-immunoreactivity overlapped with SNAPf-tagged β2 adrenergic receptor. Data are mean ± standard error of the mean. The overlapping at 0, 30, 60 min after OXT application (100 nM) was estimated to be 86.0 ± 1.1% (n = 20), 83.1 ± 1.5% (n = 18), and 85.5 ± 1.0% at 60 min (n = 20), respectively, for hOXTR-376R; 86.1 ± 1.0% (n = 23), 84.9 ± 1.6% (n = 13), 84.6 ± 1.4% (n = 20), respectively, for hOXTR-376G; and 85.2 ± 1.2% (n = 32), 84.9 ± 1.8% (n = 16), and 84.1 ± 2.2% (n = 19), respectively, for hOXTR-376C. [file 2040-2392-4-22-S2.tiff]
